# Correlations between Nutritional Status and Quality of Life of People with Parkinson’s Disease

**DOI:** 10.3390/nu15143272

**Published:** 2023-07-24

**Authors:** Raissa Dias Fernandez, Graziela Maria Benevenuto Bezerra, Lane Viana Krejcová, Daniela Lopes Gomes

**Affiliations:** 1 Neuroscience and Behaviour, Universidade Federal do Pará, Belém 66610-770, PA, Brazil; 2 Nutrition College, Universidade Federal do Pará, Belém 66610-770, PA, Brazil; graziela.bezerra@ics.ufpa.br; 3 Neuroscience and Cellular Biology, Universidade Federal do Pará, Belém 66610-710, PA, Brazil; lanekrejcova@gmail.com; 4 Human Nutrition, Universidade Federal do Pará, Belém, PA 66610-710, Brazil; danielagomes@ufpa.br

**Keywords:** Parkinson’s disease, nutritional status, quality of life

## Abstract

Parkinson’s disease (PD) is a neurodegenerative condition that can impact the nutritional status, and such impact seems to be related to the quality of life (QoL). Objective: To evaluate the correlation between anthropometric variables and the QoL of people with Parkinson’s disease (PD). Methods: This is a cross-sectional, descriptive, and analytical study, carried out through the collection of anthropometric data and application of the Parkinson’s Disease Questionnaire PDQ-39. Results: 33 individuals (23 male) diagnosed with PD participated in the research, with a mean age of 58.9 ± 11.6 years. We observed overweight in 45.4% of participants. The perception of QoL showed lower scores for the subjects in the dimensions of body discomfort (75.3 ± 16.6), social support (62.7 ± 15.7), and mobility (61.0 ± 23.6). The correlation between the total QoL score and age (model 1, B = 0.347; CI 0.004–0.902; *p* = 0.048), which remained statistically significant in the multiple linear regression, regardless of gender (model 2, B = 0.365; CI 0.016–0.937; *p* = 0.043) and BMI (model 3, B = 0.363; CI 0.006–0.943; *p* = 0.047), suggests that, in the participants of this study, this relationship does not depend on gender and nutritional status. Conclusion: The perception of QoL was worse in the dimensions of body discomfort, social support, and mobility, worsening with advanced age. Correlations between the worst scores in QoL dimensions and nutritional status were observed. A positive correlation was also identified between age and overall PDQ-39 score, regardless of gender and nutritional status.

## 1. Introduction

Parkinson’s disease (PD) is a chronic, degenerative, and progressive neurological disease that is known to affect the motor system, together with non-motor complications. It was first described in 1817 by the English physician James Parkinson [1]. The Brazilian Ministry of Health [2] states that PD is characterized by tremors at rest and in the extremities, postural instability, joint stiffness, and bradykinesia, in addition to a decreased sense of smell, sleep disturbances, alteration of intestinal rhythm, and depression. According to the World Health Organization [2], it is estimated that around 200,000 people are affected by PD in Brazil [3], being the second most frequent neurodegenerative disease among the elderly. It is common at older ages, with a peak prevalence in individuals aged between 70 and 80 years. Because it is a degenerative disease, it can cause disability 10 to 15 years after diagnosis [4].

PD has a multifactorial etiology, which includes genetic and environmental dysfunctions, among the factors that increase the risk of developing the disease, we can mention: heredity, advanced age, male gender, exposure to pesticides, head trauma (mainly from repeatedly), oxidative stress, mitochondrial abnormalities, among others [2].

Due to several changes in different neurotransmitter systems that affect general functionality, the nutritional status tends to worsen as the disease progresses. The individual with PD can present malnutrition and is very likely to lose weight due to several factors, including low nutrient intake, broad symptomatic presentation, and drug-nutrient interactions related to PD pharmacological treatment [5]. The weight loss, associated with the general symptomatic presentation aggravates the loss of autonomy and reduces the patient’s quality of life. Therefore, it is extremely important to identify nutritional changes in people with PD, with the aim of preventing malnutrition and improving the quality of life [6].

According to the Brazilian Ministry of Health [7], pharmacological treatment is recommended for symptomatic control of PD, together with adjuvant therapies that can help in slowing the progression of functional loss. In addition to this, nutritional intervention is essential for treatment to optimize dietary intake, manage the interactions between dopaminergic drugs and nutrients, reduce the side effects caused by these drugs, and attenuate nutritionally related aspects of the symptomatology of the disease [8].

The quality of life (QoL) of people living with PD can be assessed using the Parkinson’s Disease Questionnaire (PDQ-39), which comprises 39 questions regarding mobility, activities of daily living, emotional well-being, stigma, social support, cognition, communication, and bodily discomfort. Based on the answers obtained, the total score is inversely proportional to the patient’s quality of life, i.e., the lower the total score on the scale, the greater the QoL of the individual [1,9]. Each individual with PD has their own perception of their chronic health condition. The quality of life of individuals with Parkinson’s disease worsens with the duration of the disease and the progression of symptoms results in the appearance of treatment complications, with worsening in the performance of daily activities. The dimensions of quality of life that are most compromised in individuals are activities of daily living such as mobility, emotion, and physical discomfort [9].

There are currently no perspectives on a cure for Parkinson’s disease. The present treatments are carried out with the aim of delaying the prognosis and alleviating the symptoms [10,11]. The treatment of PD disease is divided into: pharmacological therapy, non-pharmacological therapy, surgical ablation, and DBS (deep brain stimulation). Pharmacological therapy is the first line of treatment, however, it can cause long-term complications such as dyskinesias The pharmacological alternatives used for the treatment of PD may include levodopa, monoamine oxidase-B (MAO-B) inhibitors, mainly rasagiline and selegiline, and catechol -Omethyltransferase (COMT), anticholinergics and dopamine agonists [11,12]. It has been also observed that, as expected, the evolution of the disease significantly influences the quality of life of the patients.

PD is a complex disease, it is necessary to speak, educate and prepare family members, caregivers, and health professionals on the subject. This is conducted in order to provide the patient with adequate care during treatment and rehabilitation. However, there are still few studies that assess the nutritional status and quality of life of these patients

A case report study [13] demonstrated that with the improvement of the patient’s nutritional status, there was also improvement in movements, recovery of activities of daily living (ADLs), and, consequently, the QoL. Therefore, the aim of the present research is to evaluate the correlation between the nutritional status evaluated through anthropometric variables and the perception of the quality of life of people with Parkinson’s Disease.

## 2. Methods

This is a cross-sectional, descriptive, and analytical study, carried out by collecting anthropometric data to identify the nutritional status of the participants and applying the Parkinson's Disease Questionnaire-39 on quality of life (PDQ-39). The data collection was carried out, in person, at the neurology outpatient clinic of Hospital Ophir Loyola (Belém-PA, Brazil), from March to May 2022. Data collection took place every Monday, in the morning, at the usual scheduled time of neurologist care. The research was carried out with 33 participants with PD, looked after by the team of the abovementioned public hospital.

This research was submitted to the Ethics Committees of the Universidade Federal do Pará (UFPA) and of the Ophir Loyola Hospital, complying with the legal requirements of Resolution 466/12, of the National Health Council. The research was approved by both committees, prot. n. 4,937,107 and 5,081,449, respectively.

All participants were diagnosed with idiopathic Parkinson’s disease, according to the criteria of the London Brain Bank (UK Parkinson’s Disease Society Brain Bank). They were individuals of both genders (23 male), at different stages of disease progression. All agreed to participate in the data collection and signed a Consent Form. If the participant had some reason that prevented him from reading and signing, the consent was read aloud and signed by a legal responsibility. Patients with indications of cognitive risk, diagnosed psychiatric illnesses, less than one year after diagnosis, and participants who refused to participate in the research or sign the informed consent form, were excluded from the study.

Data collection was performed using a structured questionnaire with closed and open questions which included the participant’s identification, age, sex, sociodemographic data with marital status, education, occupation, and average family income; as well as data on how this income was affected by the COVID-19 pandemic, if the participant received the government’s financial aid, and data regarding the social isolation carried out by the participant during this period.

The nutritional status of the participant was identified by observing the anthropometric data collected: weight, height, Body Mass Index (BMI), arm circumference (AC), and triceps cutaneous skinfold (TCS). Weight was measured using an electronic digital scale, which supported up to 180 kg. Height was measured using a portable stadiometer (200 cm, 1 cm precision). When the patient had some reason that prevented him from stepping on the scale or standing up to measure height, it was estimated according to the equations of Chumlea et al. [14].

After the data collection, the BMI of each participant was calculated (BMI = Weight/Height^2^). The classification of nutritional status through the BMI was made as recommended by the World Health Organization [15]. The other anthropometric measure taken was the triceps cutaneous skinfold (TCS), which is widely known to show a high correlation with nutritional status. For this measurement, an adipometer (lange) was used, and the measurement was performed on the posterior side of the non-dominant arm, in the skinfold right over the central body of the triceps muscle [16].

The PDQ-39 was applied to assess the quality of life of the participants. The questionnaire was divided into 8 dimensions: mobility (10 items), activities of daily living (6 items), emotional well-being (6 items), stigma (4 items), social support (3 items), cognition (4 items), communication (3 items) and body discomfort (3 items). The 39 items present in the questionnaire show 5 possible answers on a Likert scale, with scores ranging from 0 (never) to 4 (always or it is impossible for me), among them: never, from time to time, sometimes, often, always or it is impossible for me.

For each domain, the sum of the scores obtained was performed, divided by the number of questions in the domain, multiplied by 4, then by 100. The final score calculations for each participant were performed by a sum of the results from all the domains, divided by 8 (number of domains present in the PDQ-39), reaching the average score. The total score ranged from 0 (no problem) to 100 (maximum problem level). The interpretation of the results considers an inversely proportional QoL to the score obtained: the higher the score, the lower the patient’s QoL.

Statistical analysis was performed using SPSS software, version 24.0. The results of categorical variables were expressed in absolute frequency and proportion. Continuous variables were expressed in mean and standard deviation. Pearson’s correlation test was applied to evaluate bivariate correlations and statistically significant correlations were chosen to compose the multiple linear regression model. For all analyses, the statistical significance level of 95% (*p* ˂ 0.05) was considered.

## 3. Results

The mean age of the participants was 58.9 ± 11.6 years, ranging between 31 and 84 years. Of the 33 participants, the majority were male (69.7%), married (54.5%), had completed high school (24.2%), retired (63.6%), and earned from 1 to 3 minimum wage salaries monthly (60.6%). During the COVID-19 pandemic, 87.9% did not receive emergency assistance and 54.5% of the participants carried on completely the social isolation protocols (Table 1).

As for the nutritional status, the participants had a mean weight of 69.2 ± 10.4 kg, a mean BMI of 25.9 ± 3.3 kg/m^2^, and 20.0 ± 6.6 mm of TCS. Regarding the general classification of the participants’ nutritional status, excessive weight was observed in 45.4%, followed by 39.4% with eutrophy, and, to a lesser extent, 15.2% presented malnutrition/underweight (Table 2).

Regarding the dimensions of the participants’ perception of QoL, on average, the higher scores (indicative of lower QoL) were body discomfort (75.3 ± 16.6), social support (62.7 ± 15.7), mobility (61.0 ± 23.6), stigma (48.9 ± 20.2), communication (48.5 ± 18.2), emotional well-being (47.6 ± 25.6), daily physical activity (44.4 ± 20. 3) and cognition (37.4 ± 19.5), in that order. The average of the PDQ-39 for the sample was 53.2 ± 15.2, ranging from 17 to 89. Thus, an important impact on QoL is observed, especially in the domains of body discomfort, social support, and mobility (Table 3).

The correlation between age, BMI, and TCS with the different dimensions of quality of life was tested. There was a significant positive correlation between age (years) and mobility dimensions (r = 0.441; *p* = 0.005), daily physical activity (r = 0.372; *p* = 0.016), communication (r = 0.331; *p* = 0.030), body discomfort (r = 0.414; *p* = 0.008), and the total PDQ score (r = 0.347; *p* = 0.024). There was a significant positive correlation between BMI (kg/m^2^) and the dimension of social support (r = 0.648; *p* = 0.000) and a significant negative correlation between BMI (kg/m^2^) and cognition (r = −0.343; *p* = 0.025). A significant negative correlation was found between the TCS (mm) and the dimension of daily physical activity (r = −0.333; *p* = 0.029), TCS and the dimension of cognition (r = −0.374; *p* = 0.016), and a significant positive correlation between TCS and social support (r = 0.387; *p* = 0.013) (Table 4).

According to the significance indicated in the bivariate analysis and the literature, the variables for multiple linear regression were chosen. Table 5 shows the correlation between the total QoL score and age (model 1, B = 0.347; CI 0.004–0.902; *p* = 0.048), which remained statistically significant in the multiple linear regression, regardless of gender (model 2, B = 0.365; CI 0.016–0.937; *p* = 0.043) and BMI (model 3, B = 0.363; CI 0.006–0.943; *p* = 0.047), suggesting that, in the participants of this study, this relationship does not depend on the gender and nutritional status (Table 5).

## 4. Discussion

The research participants showed a low perception of QoL, with worse scores in the domains of body discomfort, social support, and mobility. Thirty-three patients with PD were evaluated, mostly male, aged over 59 years. The sex distribution of the sample corroborates epidemiological data gathered by Brazilian public health services, which shows that men are more affected by PD than women, together with higher PD prevalence in people over 50 years of age. This pattern, as the overall PD presentation worldwide, is related to the country’s aging population, as well as, possibly, to the neuroprotective effect of estrogen in women, although it affects both sexes [10,17,18]. As in the research carried out by Guerdão et al. [19], the number of married and retired people in the sample was prevalent. However, they differed in relation to the level of education. In the abovementioned study, most of the participants had completed higher education levels, meanwhile, in the present study, the majority of the sample had only completed high school.

It was also noted that during the COVID pandemic, 54.5% of the participants adhered to total social isolation, corroborating Paiva et al. [20] who concluded that the COVID-19 pandemic negatively impacted social aspects, as well as impaired the accessibility to medical centers with interruption of face-to-face visits, due to the preventive actions carried out given the high transmissibility of the SARS-CoV-2 virus, which contributed to aggravating motor and non-motor symptoms of PD. It is noteworthy that 87.9% of the individuals evaluated in this study did not receive government financial emergency aid, and financial issues can have made it difficult to obtain regular medication for PD during the period [21].

With regard to nutritional status, PD patients usually tend to be underweight or malnourished, which may be associated with increased energy expenditure caused by the progression of motor symptoms, associated with gastrointestinal symptoms and anorexia arising from the side effects of PD medications, modifying the state patient’s nutritional status [6]. Nevertheless, in the present study, we observed a different pattern in the studied population, with a higher presentation of overweight and eutrophy, corroborating previous studies [5,22,23] that obtained similar data in their analysis. It may be an effect of the PD-related reduced levels of dopamine receptors, together with the increase in neurotoxins associated with chronic inflammation, which can contribute to an excess of weight [5,24]. It is important to emphasize that body weight changes can be also associated with the therapies for Parkinson’s disease, especially during levodopa using [25]. Another possibility is that the observed pattern and nutritional status may also have been influenced by the nutritional transition experienced in Brazil in the last decades, with a general increase in overweight [15].

The present study found a worse perception of QoL in the domains of body discomfort, social support, and mobility, demonstrating that PD shows a multidimensional character [26]. In addition, the mean total score of the PDQ-39 was above 50 (53.2 ± 15.2), demonstrating a perception of low QoL by the interviewed patients. These findings corroborate a previous study [1] which also observed low QoL according to the PDQ-39.

A report published by Shalash et al. [27] that evaluated the mental health, physical activity, and quality of life of people with PD during the COVID-19 pandemic, also found that the domains of body discomfort, the most affected in the present sample, and mobility were significantly impaired, in agreement with the present research. Social support was the second worst result of the PDQ-39 domains that impacted the QoL of people with PD evaluated in this study. Foppa et al. [28], when applying the PDQ-39 in their research, also observed a lack of social support perceived in these patients, mainly due to the lack of knowledge of their social cycle regarding the limitations that PD entails, making the process of coping with the disease more difficult. In addition, other studies also demonstrated that mobility was among the three most affected domains in PD, as well as in the analyzed sample. Therefore, it is clear that functionality was significantly compromised in these individuals [1,26].

There was a correlation between age and QoL, especially the dimensions of mobility, daily physical activity, communication, and body discomfort, similar to the findings of Kanegusuku et al. [29], which concluded that both aging and the time since diagnosis of the disease are risk factors for motor impairment in these patients, due to the worsening of domains and, consequently, reduced QoL. Following the same pattern, in the sample observed in the present study, as age advanced, there was a worsening in QoL, regardless of the gender and nutritional status of the participants.

The bivariate correlation analysis showed that the lower the BMI, the more affected the cognition and the lower the social support. Thus, weight loss in PD has negative clinical and prognostic consequences, which increase the risk of morbidity and mortality [6].

It was observed that higher TCS values were correlated with lower scores (better QoL) in the dimensions of daily physical activity and cognition, that is, less impact in these domains. Nonetheless, studies show that being overweight can worsen daily physical activity and cognition, and can also induce neuro-inflammation, mainly hypothalamic, leading to a dysregulation of energy homeostasis, as well as neurodegeneration, in addition to being related to an increased risk of functional dependence, rapid motor progression in patients with PD, and cardiovascular diseases [23,30,31]. On the other hand, higher TCS values were correlated with higher social support scores, that is, this was a more affected domain. The reduced social support resulting from changes in daily life due to PD progression makes it increasingly difficult to cope with the disease since this dimension is important to increase resilience in stressful situations and the feeling of acceptance [32].

There are some limitations in the present study that must be considered when interpreting the results, such as the small sample. The study was carried out in a public hospital, which usually has more patients with lower income, the results may be affected by such socioeconomic profiles. Additionally, the less favored social strata in Brazil were known to be the most affected by the economic consequences of the COVID-19 pandemic, and such impacts must also have influenced both nutritional status and QoL results. The QoL assessment instrument applied depended on the participant’s self-report, which excludes patients with other neurodegenerative diseases that affect memory. Despite these limitations, we consider these results important to understand some factors associated with the perception of quality of life and its relationship with nutritional status in PD progression. Though, further studies with larger samples are suggested, including other variables that may impact quality of life, as well as intervention studies.

## 5. Conclusions

The people with PD in the present study were predominantly males, with a mean age above 50 years and with a prevalence of overweight. They showed a low perception of QoL, with worse scores in the domains of body discomfort, social support, and mobility. It was also observed that with the age increase, the dimensions of mobility, daily physical activity, communication, and body discomfort, were more affected, in addition to QoL as a whole. On the other hand, the lower the BMI, the more affected was cognition and social support. Higher TCS was associated with less affected dimensions of daily physical activity and cognition, however, more affected social support. A positive correlation was also identified between age and overall PDQ-39 score, regardless of gender and nutritional status. Therefore, it is essential that public policies invest in campaigns and support groups on the best ways to live with PD, and in carrying out a larger-scale mapping of these patients, in addition to offering, mainly, multidisciplinary approaches for PD management, to control the symptoms, improve the nutritional profile and the QoL of these individuals.

## Figures and Tables

**Table 1:** Socioeconomic profile of people with Parkinson’s disease followed up at a public hospital in Belém-PA, 2022.

Socioeconomic Data	*n*	%
Sex		
Male	23	69.7
Female	10	30.3
Marital status		
Single	5	15.2
Married	18	54.5
Widowed	4	12.1
Stable union	2	6.1
Divorced	4	12.1
Scholarity		
Illiterate	4	12.1
Incomplete Elementary School	2	6.1
Complete primary education	4	12.1
Incomplete high school	5	15.2
Complete high school	8	24.2
Technical education	1	3.0
Incomplete Higher Education	3	9.1
Complete Higher Education	5	15.2
Postgraduate	1	3.0
Occupation		
Retired	21	63.6
Active worker	3	9.1
Receiving government aid	9	27.3
Income		
Up to 1 minimum wage	7	21.2
>1 to 3 minimum wages	20	60.6
>3 to 6 minimum wages	6	18.2
Receiving the COVID government emergency aid		
Yes, I used it to buy medicine and food	4	12.1
I did not receive the aid	29	87.9
Adhesion to social isolation protocols		
Total isolation	18	54.5
Partial isolation	15	45.5

**Table 2:** The nutritional profile of people with Parkinson’s disease followed up at a public hospital in Belém-PA, 2022.

Nutritional Profile	Mean ± SD	Interval
Weight (Kg)	69.2 ± 10.4	52.0–97.0
Body Mass Index (BMI—kg/m^2^)	25.9 ± 3.3	18.9–32.4
Triceps Cutaneous Skinfold (TCS—mm)	20.0 ± 6.6	8.0–35.5
	N	%
BMI Classification—Adults		
Grade I malnutrition	1	3.0
Eutrophy	7	21.2
Overweight	8	24.2
Grade I obesity	3	9.1
BMI Classification—Aged		
Low weight	1	3.0
Deficit risk	3	9.1
Eutrophy	6	18.2
Overweight	4	12.1
TCS Classification		
Severe malnutrition	3	9.1
Moderate Malnutrition	3	9.1
Mild malnutrition	2	6.1
Eutrophy	6	18.2
Obesity	19	57.6

SD = Standard Deviation.

**Table 3:** Characterization of the perception of quality of life of people with Parkinson’s disease followed up at a public hospital in Belém PA, 2022.

Quality of Life Domains *	Mean ± SD	Interval
Mobility	61.0 ± 23.6	13–100
Daily Physical Activity	44.4 ± 20.3	4–96
Emotional wellbeing	47.6 ± 25.6	0–100
Estigma	48.9 ± 20.2	6–81
Social support	62.7 ± 15.7	25–92
Cognition	37.4 ± 19.5	0–94
Communication	48.5 ± 18.2	0–92
Body discomfort	75.3 ± 16.6	42–100
Total Score	53.2 ± 15.2	17–89

* SD = Standard Deviation, Parkinson Disease Questionnaire-39.

**Table 4:** Correlation between the domains of quality of life, age, and nutritional profile of people with Parkinson’s disease followed up at a public hospital in Belém-PA, 2022.

Quality of Life (QoL)	Age (Years)		BMI (kg/m^2^)		TCS (mm)	
	r	*p*-Value	R	*p*-Value	R	*p*-Value
Mobility	0.441	0.005	−0.121	0.251	−0.239	0.090
Daily Physical Activity	0.372	0.016	−0.239	0.090	−0.333	0.029
Emotional wellbeing	0.036	0.420	0.062	0.366	0.140	0.219
Estigma	0.102	0.286	0.113	0.266	0.060	0.370
Social support	0.166	0.178	0.648	0.000	0.387	0.013
Cognition	0.268	0.053	−0.343	0.025	−0.374	0.016
Communication	0.331	0.030	−0.190	0.145	−0.194	0.140
Body discomfort	0.414	0.008	−0.118	0.257	−0.044	0.404
Total score	0.347	0.024	−0.480	0.396	−0.108	0.275

Pearson correlation test.

**Table 5:** Multiple linear regression between total QoL score and age in people with subsequent Parkinson’s disease followed up at a public hospital in Belém-PA, 2022.

Total Score Quality of Life Perception *	*B*	IC 95% (Minimum; Maximum)	*p*-Value
Model 1			
Age	0.347	0.004; 0.902	0.048
Model 2			
Age	0.365	0.016; 0.937	0.043
Sex	0.109	−7.904; 14.973	0.533
Model 3			
Age	0.363	0.006; 0.943	0.047
Sex	0.120	−8.062; 15.860	0.510
Body Mass Index	−0.048	−1.890; 1.450	0.789

Multiple linear regression; Dependent variable: total PDQ score; co-variable: Age (years) and Body Mass Index (kg/m^2^), B = Regression coefficient * Parkinson Disease Questionnaire39.

## Data Availability

Data is unavailable due to privacy or ethical restrictions.
